# Risk management of sports service supply chain using fuzzy comprehensive evaluation and intelligent neural network

**DOI:** 10.1016/j.heliyon.2024.e32068

**Published:** 2024-05-28

**Authors:** Rui Cong, Fenglei Li, Lili Wang, Hailong Wang

**Affiliations:** a College of Physical Education, East China University of Technology, Nanchang, 330022, China; b East China University of Technology, Nanchang, 330022, China

**Keywords:** Sports service industry, Fuzzy comprehensive evaluation, Neural network, Supply chain risk, Questionnaire survey

## Abstract

The sports service supply chain faces various potential risks, such as market fluctuations, logistics issues, and partner uncertainties. To address these risks effectively, this study employs a combination of fuzzy comprehensive evaluation (FCE) methods and intelligent neural networks to create an innovative risk management framework. By considering diverse uncertainties and leveraging the analytical power of intelligent neural networks, this study aims to optimize the operation of the sports service supply chain and explore the risk factors within the public service supply chain of stadiums. This framework provides policy references to promote the healthy and sustainable development of the sports service industry. The main empirical findings, based on a representative survey of experts in China, are as follows: (1) When determining the weights of risk indicators for managing the public service supply chain of stadiums using the FCE method, the customer risk indicator is of paramount importance, with a weight of 0.286, accounting for 95.2 % of the total significance; and (2) In evaluating various risk indicators of the public service supply chain of stadiums through the neural network method, the customer risk indicator scores the highest, achieving a score of 76.02. Notably, the customer complaint risk indicator scores slightly higher at 79.33. Based on these findings, the study recommends focusing on enhancing customer experience within risk management strategies. Additionally, it suggests strengthening the supervision of platforms and third-party activities to ensure the stability and efficient operation of the stadium service supply chain. This study aims to provide theoretical support and reference indicators for evaluating the public service capabilities of stadiums.

## 1 Introduction

Globally, the sports service supply chain has become increasingly recognized as a vital and complex domain [1,2]. This supply chain encompasses various aspects, from strategic planning to event execution, including stadium management, ticket sales, security, food services, and other critical components [3]. However, it faces distinct challenges due to its heightened seasonality, unpredictability, and real-time demands [4]. Despite its importance, the body of research on sports service supply chains remains relatively sparse, resulting in an incomplete theoretical framework. In this context, operational research methods are essential for addressing challenges such as resource allocation, process design, and risk management within the supply chain. These methods utilize mathematical modeling and optimization technology to provide effective solutions [5,6]. Consequently, this study explores the unique challenges inherent in sports service supply chains, aiming to offer both theoretical insights and practical guidance for mitigating these challenges through the skilled application of operational research methods [7,8].

Risk management, a pivotal aspect of organizational strategy, entails the systematic identification, assessment, and mitigation of potential uncertainties and unforeseen events to minimize their adverse impacts on organizational objectives [9]. Supply chain management (SCM) represents a comprehensive strategic approach spanning the entire process from production to end-user delivery. Its overarching goals are enhancing efficiency, reducing costs, and ensuring sustainability and operational smoothness [10]. Embedded within SCM, risk management assumes a critical role in addressing potential threats and ensuring stability and resilience. Despite the widespread embrace of the service supply chain paradigm, a conspicuous void persists in the exploration of public service supply chains within the domains of stadiums and gymnasiums. This deficiency in comprehensive discourse engenders a restricted comprehension of the pivotal risk factors influencing service quality and sustainability in these environments. This uncharted terrain underscores a significant knowledge gap. To address this gap, this study concentrates on the risk management intricacies associated with the public service supply chains within stadiums and gymnasiums, aiming to investigate how risk management practices influence service quality and sustainability. Leveraging the synergistic prowess of fuzzy comprehensive evaluation (FCE) and neural network technology, this study embarks on a holistic risk assessment voyage within the service supply chains of sports stadiums. By furnishing pivotal reference indicators and offering decision support mechanisms, the study aspires to calibrate service standards and promote the sustainable evolution of the sports service industry.

This study aims to establish a theoretical framework and reference indicators to enhance the public service capabilities of stadiums, fostering industry improvement and healthy development. By integrating FCE and neural network technology, it conducts a comprehensive assessment of the risks associated with stadiums' public service supply chain, aiming to deepen understanding of management process optimization and provide targeted improvement strategies. Advancing the sports service industry in a more specialized and information-driven trajectory, this study contributes to offering higher-quality sports facility services to the public. Additionally, the integration of FCE and neural network technology introduces an innovative approach to sports service supply chain research, offering a thorough and quantitative assessment of risk factors and robust support for future enhancements and decision-making processes. The data utilized here originates from an actual questionnaire survey, marking a pioneering contribution in this research area and paving the way for further studies in sports service SCM through the introduction of novel methods and tools.

## 2 Literature review

Viglia and Dolnicar [11] found that the fuzzy comprehensive evaluation method could effectively assess uncertainty risks in the sports service supply chain. They proposed a method that combined qualitative and quantitative analysis, offering new perspectives and tools for risk management. Choi [12] categorized governmental functions into distinct domains, including allocation, development, utilization, and control of personnel, resources, and information. Current research on service supply chains primarily focuses on key areas such as tourism, port services, hotel services, and financial services. Sørensen et al. [13] applied intelligent neural networks to study risk prediction models in the sports service supply chain. Their research demonstrated that deep learning algorithms could significantly enhance the accuracy of risk prediction and provide robust support for real-time risk management. Williams et al. [14] discussed the integrated application of fuzzy comprehensive evaluation and intelligent neural networks in the risk management of the sports service supply chain. Their research indicated that this integrated method could more comprehensively identify and evaluate risks, thereby improving the overall resilience and responsiveness of SCM.

Furthermore, stadiums and gymnasiums play a pivotal role in the sports service supply chain, making research on their public service capabilities both distinctive and essential. Prior studies highlight that these stadiums extend beyond being mere locations for competitions and entertainment; they serve as central hubs for community activities and sports events. For instance, Nazari-Shirkouhi et al. [15] extensively examined stadium operation and management, revealing that operational decisions directly impact audience experience quality. Factors such as seating arrangements, safety measures, and convenience facilities significantly enhance audience satisfaction, increasing the appeal of sports stadiums and fostering audience loyalty. Wu et al. [16] identified a significant correlation between the public service level of stadiums, city image, and tourism development. Their research emphasized the strategic potential of stadiums in urban planning, considering them integral to urban development strategies. By hosting various sporting events and cultural activities, stadiums attract more tourists, improve city visibility, and contribute to economic and social benefits [17].

Furthermore, operations research methods have been utilized both domestically and internationally to study the sports service supply chain. For example, Wang and Wang [18] explored the impact of risks from third-party service providers on the stability of stadium service supply chains. Their findings highlighted the significant risks posed by improper operations or instability from these providers, recommending enhanced supervision and management. Their case study of the China Basketball Club provided valuable practical insights into sports service SCM. Sawaean and Ali [19] focused on information orientation, examining the effects of personnel, technology, and economic benefits on organizational performance, particularly in decision-making and management. This research underscored the importance of information processing in risk analysis and the flow of information within the sports service supply chain. Moeini and Rivard [20] studied the role of risk attitude in organization and project management, offering a practical framework and tools to help managers understand, measure, and respond to risks. Their work proved valuable for risk analysis, particularly within the unique context of the sports service supply chain. Cui et al. [21] concentrated on operations management, including sustainability and SCM. By exploring core concepts such as production, inventory, and distribution, they established a theoretical foundation for studying sports service supply chain operations. Their research provided a comprehensive reference for scholars interested in sustainability and SCM.

While numerous studies have explored public service capability, stadium public service capability, and service supply chains in China and other countries, Chinese scholars have primarily focused on developing theoretical frameworks for assessing public service capability. Building upon this foundation, this study introduces service supply chain theory to the stadium service industry, facilitating the evaluation of stadium public service capabilities. This approach simplifies the evaluation process, enhances operability, improves the public service capabilities of stadiums, and optimizes the allocation of public service resources in stadiums and gymnasiums.

## 3 Research methodology

To ensure the completeness and accuracy of the comprehensive risk assessment of the stadium supply service chain, FCE, the single-factor evaluation matrix, and weighted indicators are chosen based on several considerations and scientific rationale. Firstly, in many practical situations, risk factors often lack clear definitions and involve varying degrees of fuzziness and uncertainty. FCE methods excel in handling such fuzziness by accommodating uncertain input information, such as fuzzy concepts or partially true data, thereby yielding more realistic assessment results. Additionally, when assessing the risks of complex systems, it is necessary to consider the influence of multiple factors. FCE methods adeptly integrate these factors, comprehensively considering each factor's effects through fuzzy mathematical calculations, thus obtaining more thorough risk assessment results. The FCE method is instrumental in offering a detailed risk assessment that accounts for various uncertainties. Secondly, the single-factor evaluation matrix method assesses each factor within the stadium service supply chain individually. This approach helps identify which factors have a more substantial influence on overall risk, facilitating the implementation of targeted risk management measures [22]. It also provides insights into the relative importance of each factor, enabling a better understanding of risk origins. Finally, the weighted indicator method combines the weights of multiple factors to calculate a comprehensive risk score through weighted summation. This method considers various factors' relative contributions, thus contributing to a more complete risk assessment. By integrating these methods, the assessment process becomes more robust, addressing the multifaceted nature of risks within the stadium supply service chain.

### 3.1 Stadium service supply chain

The sports stadium service supply chain encompasses a comprehensive process from planning to the operational phases of stadiums. It covers various aspects such as public services, event preparation, ticket sales, stadium management, and security [23]. The primary objective is to efficiently organize and manage stadiums to meet the diverse requirements of different sports events, ensuring that the public can enjoy high-quality, safe, and seamless sports services. After major events, the service supply chain faces challenges related to the rational utilization of facilities, enhancing service capabilities, and meeting societal demands for stadium services.

(1) Analysis of the current public service capacity of stadiums

Stadiums, as central hubs for public sports services and sports development, play a pivotal role in advancing national fitness and supporting the establishment of a robust sports-oriented nation. In preparation for large-scale sports events, various provinces have constructed numerous expansive stadiums to accommodate these events' requirements. However, post-events, these large stadiums often face challenges due to high operating costs and limited service capacities, leading to underutilization and restricted public access [24]. Concurrently, there is a shortage of privately owned stadiums to meet societal demands, necessitating solutions to address these issues.

(2) Structure of stadium service supply chain

The supply chain structure of stadium services is outlined in Table 1 [25].

(3) Feasibility of introducing service supply chain theory into stadium services
  1) Government support

**Table 1:** Stadium service supply chain structure.

Structure division	Specific content
Stadium service provider	They offer specialized fitness services for children, teenagers, and older adults. Suppliers are divided based on the diverse needs of different groups, including youth training providers and elderly physique monitoring providers. The government also acts as a public service provider, offering policy and financial support.
Stadium service integrator	Stadiums serve as intermediaries between service suppliers and consumers, managing and coordinating stadium services throughout the supply chain. They implement intensive management and set service standards to provide efficient and convenient services for citizens.
Stadium service demanders	The demanders include everyone. Special green channels for ID card verification are established in project stadiums to streamline processes, especially for those who find using mobile phones and computers inconvenient.

Note: This table is compiled and collected from reference.

Note: The data comes from reference [25].

Efforts to establish a foundational public sports service system involve comprehensive plans for constructing universally accessible fitness facilities. This initiative includes creating sports parks, community fitness centers, and other facilities, aiming to ensure convenient access to suitable stadiums for individuals [26]. Concurrently, the development of urban jogging trails and greenways is promoted to achieve a "15-min fitness circle" encompassing the populace, providing accessible locations for those seeking exercise opportunities. Encouraging social forces to organize or participate in the management and operation of sports facilities further underscores the commitment to fostering widespread physical activity. Additionally, the government has enacted legislation, regulations, policy guidance, and preferential measures within the stadium service industry [27]. These measures address the shortage of stadiums and regulate the industry's development.

2) The social stadium service industry chain has taken shape

The social stadium service industry chain has taken shape, encompassing various sectors beyond traditional domains such as fitness and physical fitness monitoring [28]. An increasing number of industries, including transportation, real estate, hotels, insurance, and investment, have begun participating in developing this industry, introducing a range of fitness programs and equipment. This diversification has laid the foundation for the emergence of the stadium service supply chain and providers [29]. Additionally, numerous prominent enterprises and social organizations have joined the supply chain of stadiums and gymnasiums, contributing to its gradual expansion [30]. Leveraging rapid advancements in information technology, there is substantial potential to create a specialized information platform for fitness enthusiasts. This platform would cater to individuals seeking sports facilities and fitness services, offering robust technical support for integrators. With a considerable and rapidly growing population of fitness enthusiasts, the market exhibits significant potential as service providers, integrators, and demanders are beginning to take concrete form, thus bolstering the development of the service supply chain [31].

### 3.2 Risk of stadium service supply chain based on an FCE

The risk in the sports stadium service chain encompasses various uncertainties and potential threats that may arise throughout the entire operational process of stadiums. These risks cover government services, platform operations, sports facility projects, third-party collaborations, and customer complaints [32].

(1) Service supply chain theory

Service supply chain theory conceptualizes all affiliated enterprises within the supply chain as a unified entity [33]. It emphasizes a coordination mechanism based on cooperation and competition to ensure the integration and distributed operations of enterprises. This approach incorporates various advanced manufacturing and management technologies, utilizing information technology to support and seamlessly combine internal and external facets of enterprises. This facilitates integrated management and ensures fast and efficient service delivery. The goal is to achieve global optimization of the supply chain by considering global manufacturing resources as optional objects. Core theories within SCM include.

(1) System Management and Global Optimization: Managing the entire supply chain as a single system to achieve the best overall performance.
(2) Complementary Advantages: Leveraging the strengths of different entities within the supply chain.
(3) Customer Orientation: Focusing on fulfilling customer needs and expectations.
(4) Advanced Management Technology: Utilizing cutting-edge technologies and practices for improved management and operations.

**Fig. 1:**
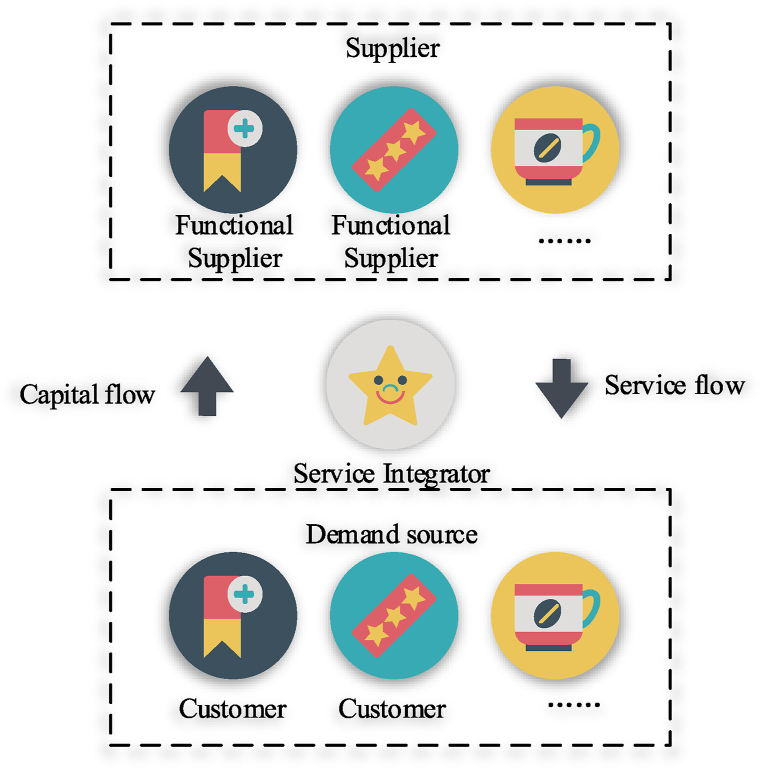
Structure of service supply chains. Note: The data comes from reference [34].

The foundational model of the service supply chain is illustrated in Fig. 1.

(2) Supply chain risk management (SCRM)

SCRM focuses on identifying and assessing various internal and external risk factors within the supply chain. Specific management measures are then employed to control and mitigate these risks. This process permeates the operational framework of the enterprise's supply chain, encompassing all dimensions and sectors [35].

(3) Risk evaluation indicator system of stadium service supply chain

To refine the risk assessment framework, a comprehensive approach is adopted. This involves reviewing relevant literature and policy documents, conducting on-site assessments of stadium operations, and integrating findings from questionnaire surveys and expert evaluations. These indicators are meticulously classified, integrated, and modified before their application [36]. Ultimately, a set of five primary indicators and nine secondary indicators is devised for assessing the risk of the stadium service supply chain. The detailed content of these indicators is outlined in Table 2.

(4) FCE of supply chain risk of stadium service

**Table 2:** Risk evaluation indicator system of stadium service supply chain.

Primary indicator	Second indicator
Government service risk	Government resource utilization risk
Project risk of the stadium	Stadium supply risk
	Project supervision risk
Platform risk	Platform construction risk
	Platform information processing risk
	Platform operation risk
Third-party risk	Third-party supervision risk
Customer risk	Equipment use risk
	Customer complaint risk

Note: The data is sourced from the research articles.

Due to the inherent uncertainty of risks and the intricate and dynamic nature of supply chain factors in stadium services, achieving precise quantitative descriptions of risk factors is unattainable. Consequently, this study employs a comprehensive evaluation approach based on the steps outlined in the FCE method to assess the risk indicators within the stadium service supply chain [37].

The steps of FCE are as follows:

Given factor set $U=\left\{u_{1},u_{2},\ldots ,u_{n}\right\}$ and evaluation set $V=\left\{v_{1},v_{2},\ldots ,v_{n}\right\}$. Weight assignments for each factor are represented by $Q=\left\{q_{1},q_{2},\ldots ,q_{n}\right\}$, where $q_{i}$ is the weight corresponding to the *i*-th factor $u_{i}$. *Q* is a fuzzy subset on *U,* as in Equation (1):

(1) $$\sum_{i=1}^{n}q_{i}=1\left(q_{i}\geq 0\right)$$

The fuzzy assessment for the *i*-th factor is a fuzzy subset on V, denoted as $R_{i}=\{r_{i1},r_{i2},\ldots ,r_{im}$}. The assessment matrix *R* is expressed as Equation (2):

(2) $$R=\left[\begin{array}{ccc} r_{11} & \ldots & r_{1m}\\ \ldots & \ldots & \ldots \\ r_{n1} & \ldots & r_{mn} \end{array}\right]$$

The judgment result *B* is a fuzzy set on the set *V*, represented as *B=V*. Results obtained through fuzzy comprehensive judgment generally cannot definitively affirm or negate their belonging to a specific level of judgment. Instead, they indicate the degree of membership across various levels. Therefore, employing the principle of maximum membership is necessary to derive a more intuitive explanation and clear judgment.

### 3.3 Risk of sports stadium service supply chain based on neural network and FCE

(1) Basic working principle of neural network

The core operations of neural networks revolve around learning and execution. Through the learning process, neural networks acquire heightened sensitivity to specific information patterns. During execution, they discern relevant information patterns or features [38].

Artificial neural networks offer several key advantages, including high classification accuracy, robust distributed processing capabilities, efficient storage and learning, strong resistance to interference and fault tolerance, and outstanding associative memory function. However, notable drawbacks include the need for massive parameters, the inherent challenge of intuitively observing the learning process, difficulty interpreting output results, and prolonged learning time. Inadequate sample sizes may even impede the achievement of intended learning objectives [39].

These drawbacks impact credibility and hinder the effective application of the approach. Additionally, the learning process is time-consuming, and insufficient sample sizes may hinder the attainment of learning objectives.

(2) Construction of sports service chain risk assessment model based on back propagation neural network (BPNN) and fuzzy evaluation

FCE involves establishing the judgment matrix R and weights Q through expert scoring, followed by the calculation of the judgment result B [40]. However, this method is subjective and inefficient. Therefore, the experiment proposes a neural network-based FCE method, specifically utilizing the Convolutional Neural Network (CNN) to address challenges associated with fuzzy evaluation. The calculation of the BPNN is expressed in Equations (3), (4):

(3) $$S_{j}=\sum_{i=0}^{n}W_{ji}∙X_{i}+b_{j}$$

(4) $$Y_{j}=f\left(S_{i}\right)$$

Here, $X_{i}$ and $Y_{j}$ represent input and output values; $b_{j}$ refers to the bias; $W_{ji}$ indicates the connection weight coefficient; *f* denotes the transformation function [41].

1) Design of CNN

**Fig. 2:**
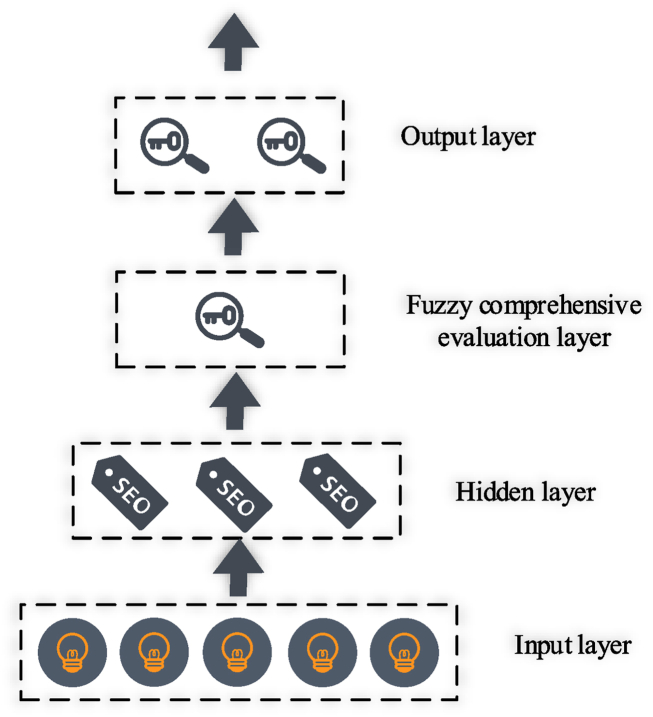
Schematic diagram of neural network with a hidden layer. Note: The data is sourced from the research articles.

The BPNN, employing the error backpropagation algorithm, is a multi-layer feedforward network extensively applied in current research. The entire information transmission process follows a unidirectional path from the input layer to the output layer. During training, the connection strengths between neurons are iteratively adjusted to minimize the disparity between the calculated output dependent variable vector of the network and the known dependent variable vector from the training samples, as depicted in Fig. 2.

In Fig. 2, the constructed BPNN integrates the FCE method, enabling it to generate fuzzy evaluation results. This network structure consists of several layers. Input layers receive input data from fuzzy evaluations. The input data undergoes processing through convolutional layers, where feature extraction occurs. Activation functions introduce non-linearity into the network, enabling it to learn complex patterns. FCE layers allow for the incorporation of fuzzy evaluation principles. Fully connected layers map the extracted features to the output layer. The output layers generate comprehensive results for the fuzzy evaluation. This integrated structure enables the BPNN to effectively handle fuzzy evaluation tasks by processing input data, extracting relevant features, and producing fuzzy evaluation results through the output layer.

2) Process Construction

**Fig. 3:**
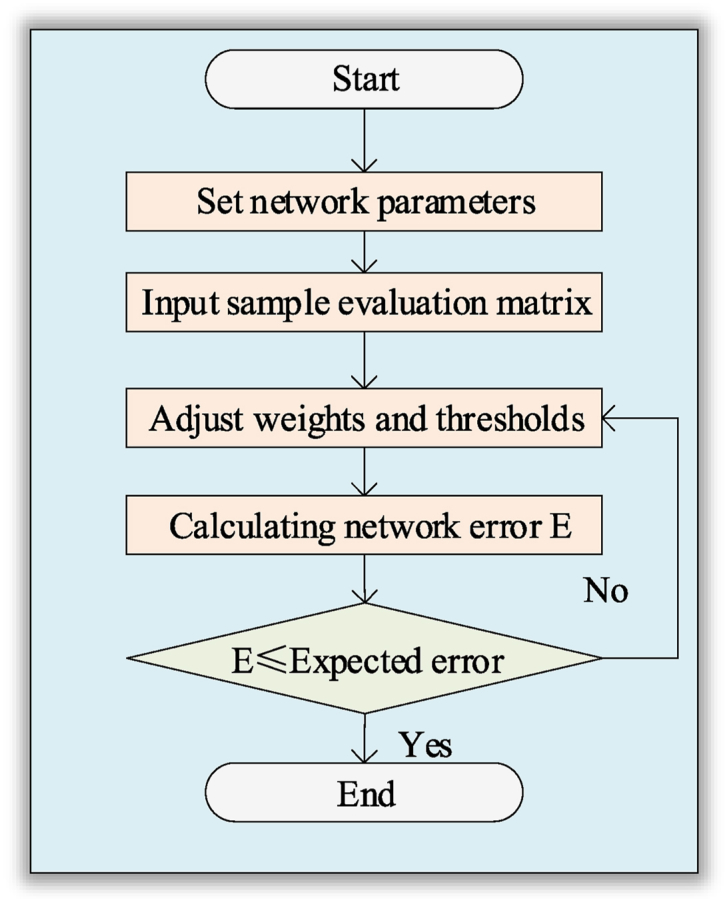
Model flow Note: The data is sourced from the research articles.

The flow of the sports service chain risk assessment model, based on the BPNN and fuzzy evaluation method constructed in this study, is illustrated in Fig. 3.

In Fig. 3, the constructed BPNN integrates the FCE method, rendering it suitable for outputting fuzzy evaluation results. This network structure comprises an input layer to receive input data from fuzzy evaluations. Subsequently, the data undergoes processing through convolutional layers, activation functions, and the FCE layer, effectively extracting feature information from the input. These features are mapped to the output layer through the fully connected layer, ultimately generating a comprehensive result for the fuzzy evaluation. This comprehensive model enables the CNN to automatically capture and comprehend the fuzzy evaluation relationships among various indicators, facilitating effective classification and assessment of input samples.

3) Parameter design

Considering the content of the sports service evaluation system and the structure of the BPNN, the designed network parameters are outlined in Table 3.

**Table 3:** Network parameter design.

Parameter name	Describe
Input features	Government service risks, stadium project risks, platform risks, third-party risks, and customer risks
Input dimensions	5
Number of convolutional layer units	9
Number of layers	4
Activation function	Sigmoid
Number of FCE layer units	5
Number of output units	1 (indicates comprehensive evaluation value)
Output activation function	Identity
Optimization	Adam
Learning rate	0.001
Batch size	12
Training rounds	100
Error function	MeanSquaredError
Data preprocessing	normalization, forwarding

Note: The data is sourced from the experimental environment parameter settings.

## 4 Experimental design and performance evaluation

### 4.1 Datasets collection

(1) Questionnaire survey

The questionnaire scale utilized here comprehensively covers the risk assessment indicator system of the public service supply chain in stadiums. The questions are meticulously designed to present various characteristics and diversities in detail. This analysis uses a 5-point Likert scale ("Excellent", "Good", "Adequate", "Poor", and "Very Poor"). By quantitatively assessing five primary indicators, namely government services, sports facility projects, platform risks, third-party risks, and customer risks: this study delves into the multidimensional features of the sports service supply chain. Furthermore, breaking down each primary indicator into secondary indicators, such as government resource utilization risk, sports facility supply risk, project supervision risk, etc., further highlights the diversity and comprehensiveness of the survey.

In November 2021, a survey using the questionnaire was conducted in several sports stadiums within a city. 200 questionnaires were distributed, with 185 recovered, yielding a recovery rate of 92.50 %. Three questionnaires were incomplete, leaving 183 valid questionnaires and achieving a response rate of 98.9 %. These 183 valid questionnaires serve as the foundational data for this study.

(2) Distribution of questionnaires

Regarding the selection of survey subjects, this study primarily focuses on managers and individuals responsible for stadium supply to ensure that respondents possess relevant professional knowledge and experience. To secure a representative sample, random sampling was employed, and the data from 183 valid questionnaires were analyzed. Preliminary analysis revealed that among the survey participants, there were a total of 100 males and 83 females, aged between 28 and 46. Among them, 90 individuals held management positions, while 93 were in service-related roles within the field. Additionally, 5 experts in risk management, consisting of 3 males and 2 females, participated in the survey.

The choice of a sample size of 200 questionnaires is based on statistical considerations. Larger sample sizes typically yield higher statistical power, facilitating more accurate detection of potential correlations or effects in the research. Moreover, a larger sample can better represent the entire management and responsible group of stadium suppliers, thereby enhancing this study's external validity. This decision adheres to statistical principles, ensuring this study obtains reliable and representative data, thus bolstering the research results' scientific rigor and credibility.

(3) Questionnaire reliability analysis

A questionnaire was formulated after establishing the evaluation indicator system for the public service capacity of stadiums and gymnasiums based on the service supply chain. To ensure the questionnaire's validity, a reliability analysis was conducted, typically using Cronbach's reliability coefficient for evaluation. According to academic experts, when Cronbach's coefficient exceeds 0.65, it can be considered that the questionnaire is consistent, making the research results adaptable and reliable. In the reliability analysis of this study, Cronbach's reliability coefficient reaches 0.79 for risk assessment of the public service supply chain in stadiums. This exceeds the standard coefficient value, indicating that the scale is effective in statistical analysis of questionnaire data and is suitable for use in such studies.

### 4.2 Experimental environment

The experimental environment utilizes the SPSS software to conduct Kaiser-Meyer-Olkin (KMO) measurement and Bartlett's test of sphericity using data acquired from the questionnaire. The KMO serves as a measure of sampling adequacy, with values ranging from 0 to 1. A higher value, approaching 1, indicates greater commonality among variables, lower net correlation coefficients among variables, and better suitability for factor analysis. Meanwhile, Bartlett's test examines whether the correlation matrix is an identity matrix. A significance level below 0.05 rejects the null hypothesis and accepts the hypothesis suggesting the presence of common factors among the correlation matrices representing the entire population, making it more conducive to factor analysis.

### 4.3 Parameters setting

To enhance the description and analysis of data output, this study designs the parameters listed in Table 4 as risk evaluation indicators for stadium service supply chains.

**Table 4:** Setting of risk evaluation indicator parameters of stadium service supply chain.

Primary indicator	Parameter setting	Secondary indicator	Parameter setting
Government service risk	H1	Government resource utilization risk	H11
Project risk of the stadium	H2	Stadium supply risk	H21
		Project supervision risk	H22
Platform risk	H3	Platform construction risk	H31
		Platform information processing risk	H32
		Platform operation risk	H33
Third-party risk	H4	Third-party supervision risk	H41
Customer risk	H5	Equipment use risk	H51
		Customer complaint risk	H52

Note: The data is sourced from the research articles.

### 4.4 Performance evaluation

(1) Results of reliability and validity test and descriptive analysis of the questionnaire

Using the SPSS software, the data extracted from the questionnaire underwent analysis, including KMO measurement, Bartlett's sphericity test, and Cronbach's alpha coefficient analysis. The outcomes of these analyses are listed in Table 5.

**Table 5:** Test results of reliability and validity of questionnaire.

KMO measurement of sampling adequacy		0.827
Bartlett's sphericity test	Approximate chi-square value	1824.74
	df	351
	Sig.	0
Cronbach's alpha coefficient		0.849

Note: The data is sourced from the questionnaire analysis results.

Table 5 indicates that the KMO measurement value for each scale within the questionnaire is 0.827. As for Bartlett's sphericity test outcomes for each scale in the questionnaire, the approximate chi-square value is 1824.74, with a degree of freedom (df) value of 351, and a significance (Sig.) value of 0. These findings confirm that the designed questionnaire exhibits commendable reliability and validity. Furthermore, the Cronbach's alpha coefficient of the questionnaire is 0.849, which exceeds 0.8, illustrating a certain level of reliability.

Through a comprehensive analysis of nationwide sports stadium construction and operation projects, the experiment involves a total of 150 stadium projects. Descriptive statistical information on risk factors is obtained by investigating and collecting data on various areas such as government resource utilization, supply chain, project supervision, platform facility construction, information processing, operational risks, third-party supervision, equipment usage, and customer complaints. The objective is to provide valuable risk management references for project managers and decision-makers. The descriptive statistics for the risk factors outlined in this study are presented in Table 6.

**Table 6:** Descriptive statistics of risk factors.

Risk factor	Mean value	Standard deviation (SD)	Significance	Degree of freedom
Risk of government resource utilization	3.42	0.76	0	301
Supply risk of stadium projects	2.91	0.68	0	27
Project supervision risk	3.15	0.72	0	221
Platform facilities construction risk	2.78	0.64	0	210
Platform information processing risk	2.96	0.71	0	178
Platform operation risk	3.08	0.69	0	399
Third-party supervision risk	2.99	0.67	0	354
Equipment use risk	3.25	0.75	0	207
Customer complaint risk	3.12	0.73	0	43

Note: Comprehensive analysis of 150 stadiums in China.

Table 6 provides descriptive statistics on various risk factors discussed here, encompassing mean values and standard deviations (SD). These statistics offer crucial insights into the distribution and variability of the specified risk factors. Analyzing the data from Table 6 reveals that the mean value for government resource utilization risk (mean: 3.42, SD: 0.76) in the sample is comparatively high, indicating notable dispersion and uncertainty in the utilization of government resources. Conversely, the mean value for the supply risk (mean: 2.91, SD: 0.68) associated with stadium projects is low, with a relatively small SD, suggesting a consistent distribution of this risk factor in the sample. Moreover, the mean value for project supervision risk (mean: 3.15, SD: 0.72) falls within a moderate range, while the relatively large SD implies significant variability in project supervision.

The research findings reveal that government resource utilization risk exhibits a relatively high mean value within the sample, accompanied by significant dispersion. This suggests potential uncertainty and variability in the utilization of government resources. In contrast, the mean value for the supply risk associated with stadium projects is comparatively low, with a relatively consistent distribution. Meanwhile, project supervision risk falls within a moderate range yet displays notable variability.

(2) FCE results of supply chain risk of stadium public service

The risk indicator weight of the stadium service supply chain is determined using the FCE method. "The importance ratio (%)" refers to the relative importance of each risk indicator in the risk assessment of the stadium service supply chain. Specifically, this ratio represents the proportion of each risk indicator weight in the overall assessment when using the FCE method for risk assessment. It reflects the impact of different risk factors on the stability and efficiency of the stadium service supply chain, with a higher ratio indicating that the risk factor is more critical in overall risk management. The outcomes are shown in Table 7.

**Table 7:** Calculation results of indicator weight of level 1.

Primary indicator	Weight	The importance ratio (%)
Government service risk H1	0	0
Project risk of stadium H2	0.052	17.7
Platform risk H3	0.224	76.5
Third-party risk H4	0.276	94.1
Customer risk H5	0.286	95.2

Note: The data is sourced from the experimental results analysis.

Table 7 presents the evaluation of stadium service supply chain risk using the FCE method. The government service risk indicator is assigned a weight value and importance ratio of 0, indicating minimal influence. In contrast, the stadium risk indicators hold a weight value of 0.052, representing 17.7 % importance. The platform risk indicators are assigned a weight value of 0.224, constituting 76.5 % importance. The third-party risk indicator carries a weight of 0.276, accounting for 94.1 % of its importance. Notably, the customer risk indicator holds the highest weight of 0.286, making up 95.2 % of its importance. These results underscore the significant impact of the customer risk indicator on the overall risk of the public service supply chain in stadiums.

The research outcomes indicate that the government service risk indicator has minimal impact, whereas the customer risk indicator, specifically focusing on customer satisfaction, significantly influences the sports stadium service supply chain. Consequently, optimizing strategies should prioritize addressing customer-related risks to enhance overall service quality and effectiveness.

(3) Results of risk assessment for the public service supply chain of sports stadiums based on FCE method
  1) Score results for primary indicators

**Fig. 4:**
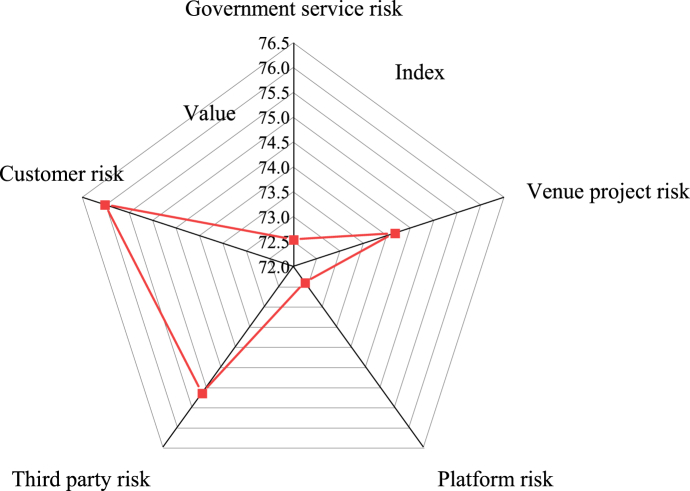
Weighting results of primary risk indicators of the public service supply chain of stadiums. Note: The data is sourced from the experimental results analysis.

Using a comprehensive evaluation model based on the BPNN algorithm and FCE method to analyze questionnaire data, scores for primary risk indicators in the public service supply chain of sports stadiums are obtained. These scores are illustrated in Fig. 4.

Fig. 4 illustrates the assessment results of primary risk indicators in the stadium service supply chain using the FCE method. The government service risk indicator attains a score of 72.54, while the stadium risk indicator scores 74.16. Additionally, the platform and third-party risk indicators reach scores of 72.4 and 75.15, respectively. Notably, the customer risk indicator garners the highest score among all indicators influencing the risk of the public service supply chain of stadiums, consistent with the FCE outcome.

2) Grade results of the second indicator

**Fig. 5:**
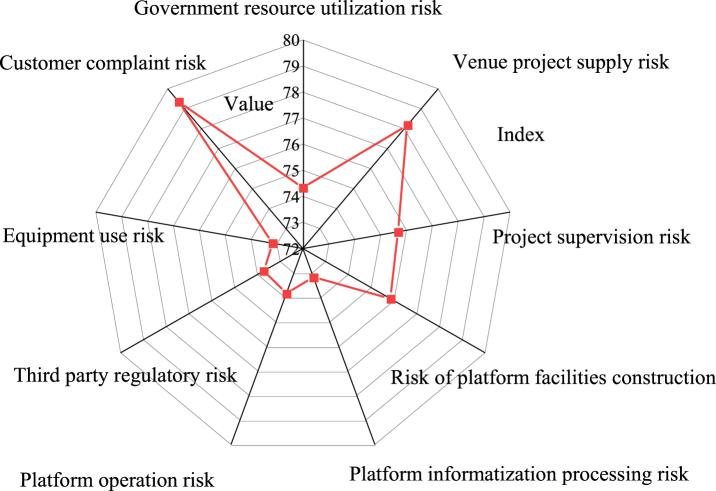
Weighting results of secondary risk indicators in the public service supply chain of stadiums Note: The data is sourced from the experimental results analysis.

Utilizing the comprehensive evaluation model based on the BPNN algorithm and FCE method, Fig. 5 illustrates the scores obtained for the secondary risk indicators of the public service supply chain of stadiums.

Fig. 5 illustrates the scores obtained for the secondary risk indicators of the public service supply chain of stadiums using the FCE method. The score values for the risk indicators are as follows: 74.33 for government resource utilization, 78.17 for stadium supply risk, 75.67 for project supervision risk, 75.84 for platform construction risk, 73.17 for platform information processing risk, and 73.83 for platform operation risk. Additionally, the third-party supervision, equipment use, and customer complaint risk indicators score 73.72, 73.17, and 79.33, respectively. This data underscores that among the secondary risk indicators of the public service supply chain in stadiums, the customer complaint risk indicator attains the highest score, consistent with the primary risk indicator score.

3) Comparative analysis with expert evaluation results

**Fig. 6:**
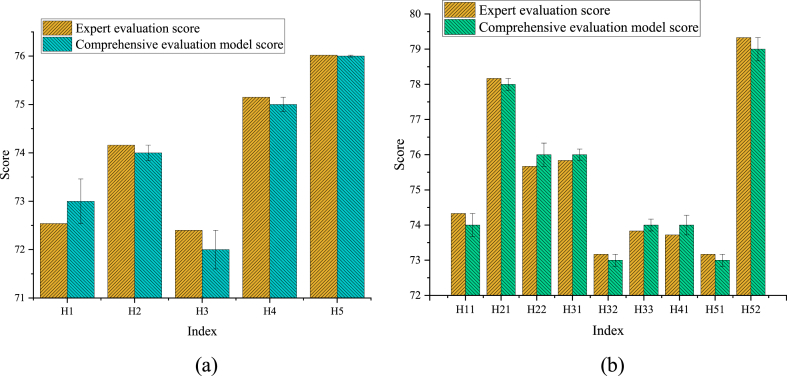
Comparative evaluation results (a) Comparison of evaluation results for primary indicators; (b) Comparison of evaluation results for secondary indicators Note: The data is sourced from the experimental results analysis.

The obtained evaluation results are compared with the evaluation results from expert assessments, and the results are revealed in Fig. 6a and b.

Fig. 6a and b illustrate that the scores obtained from the FCE model closely align with those from expert evaluations, with variances ranging from 0.02 to 0.46 for primary indicators. This suggests that the FCE model accurately reflects expert opinions in the overall assessment of the sports service supply chain risk, with minimal differences in scores among various indicators. Similarly, the scores for secondary indicators from the proposed model exhibit small differences compared to expert evaluations, mainly ranging from 0.16 to 0.33. These findings demonstrate the proposed model's consistency with expert evaluation in assessing risk factors in a more specific and detailed manner, showcasing its practicality and credibility. Overall, the comparative evaluation results in Fig. 6 show that the FCE model performs well in both primary and secondary indicator assessments, demonstrating consistency with expert evaluation results. This denotes that the model can provide reliable outcomes for sports service supply chain risk assessments, accurately reflecting expert opinions across different indicators.

### 4.5 Discussion

This study utilizes the FCE method to assess the risk of the stadium service supply chain. Specifically, the risk associated with utilizing government funds exhibits a relatively high mean value, coupled with a considerable standard deviation. This suggests that achieving effective utilization of government resources may encounter substantial challenges. Conversely, despite the low average supply risk observed in stadium projects, the relatively small standard deviation indicates a more consistent distribution of supply risks. These findings provide a fundamental framework for prioritizing and distributing resources among various risk factors. In contrast to earlier studies, this study emphasizes customer risk within the supply chain risk framework of stadiums. This aligns with the findings of Ge et al. [42], highlighting the pivotal role of customer satisfaction in the successful operational dynamics of stadiums. However, unlike the work by Regin et al. [43], this study reveals a higher average for the risk associated with government resource utilization, potentially attributed to specific influencing factors within particular regions or stadiums. Such comparative analyses are instrumental in identifying disparities between studies and guiding future research directions. Furthermore, this study assigns the highest weight to customer risk when employing the FCE method to evaluate the risk of the stadium service supply chain. This result aligns with the operational realities of sports stadiums, where a commitment to delivering high-quality services is paramount for attracting and retaining customers. Consumer satisfaction is thus considered a critical objective, consistent with the viewpoint of Pham et al. [44], who advocate prioritizing consumers' needs in SCRM. The primary and secondary indicator scores obtained through a comprehensive neural network evaluation underscore the significance of customer satisfaction in the public service supply chain of stadiums and gymnasiums. However, this study also underscores the presence of government service risk and platform risk, both exerting an influence on the supply chain's stability to a certain extent [45].

Furthermore, according to the research findings, the customer risk indicator holds the greatest weight in the risk assessment of the public service supply chain in stadiums. This outcome underscores the critical role of customer risk in influencing overall stability and sustainability within the service supply chain [46]. Indeed, as the primary beneficiaries in the service chain, customers directly impact operational effectiveness through their demands, satisfaction levels, and grievances. The significant weight assigned to the customer risk indicator implies a necessity for heightened attention and optimization in managing aspects related to customer relationships. Possible issues may include service quality, equipment usage experience, and timely response to and resolution of customer complaints.

## 5 Conclusion

This study utilizes a comprehensive fuzzy assessment and intelligent neural networks to analyze the risk assessment of the supply chain for stadium services. The research findings suggest that in formulating risk management strategies, the government should prioritize enhancing customer information security and strengthening supervision of platforms and third-party activities to ensure the security of the sports stadium service supply chain. By employing FCE and intelligent neural network assistance, this study addresses critical issues within this realm. The survey results highlight multi-level risks in government service, stadium projects, platforms, third parties, and customers, with the highest weight assigned to customer risk. This indicates the need for increased attention and consideration of the impact of customer satisfaction throughout the management of the stadium services supply chain. The application of assessment methods with neural networks enables a more comprehensive representation of the complexity of risks and reveals correlations between different factors. However, this study has some limitations. Firstly, the research scope is limited to a few selected stadium projects, potentially leading to a lack of representativeness in the data selection. To gain a more comprehensive understanding of the risks in the stadium service supply chain, future research could expand the scope to cover more projects and stadium types. Secondly, the model construction and evaluation methods still require further improvement and validation to enhance methodological credibility. Future studies could employ more advanced methods and tools to deepen understanding of risks in the stadium service supply chain. Additionally, to bolster external validity, consideration could be given to multiple cases spanning different regions and cultural backgrounds to obtain more extensive and representative data. Consequently, this approach can offer more targeted management recommendations for the entire stadium service industry.

## Data availability statement

Data will be made available on request.

## CRediT authorship contribution statement

**Rui Cong:** Writing – original draft, Methodology, Investigation, Formal analysis, Data curation. **Fenglei Li:** Writing – review & editing, Validation, Supervision, Software. **Lili Wang:** Software, Resources, Methodology, Formal analysis. **Hailong Wang:** Visualization, Software, Methodology.

## Declaration of competing interest

The authors declare that they have no known competing financial interests or personal relationships that could have appeared to influence the work reported in this paper.

## References

[1] Jiang M., Lu J., Qu Z., Yang Z. (2021). Port vulnerability assessment from a supply chain perspective. Ocean Coast Manag..

[2] Creazza A., Colicchia C., Spiezia S., Dallari F. (2022). Who cares? Supply chain managers' perceptions regarding cyber supply chain risk management in the digital transformation era. Supply Chain Manag.: Int. J..

[3] Zhang D., Tang Y., Yan X. (2023). Supply chain risk management of badminton supplies company using decision tree model assisted by fuzzy comprehensive evaluation. Expet Syst..

[4] Li J.A. (2023). Fuzzy comprehensive evaluation method of regional economic development quality based on a convolutional neural network. J. Circ. Syst. Comput..

[5] Haque M., Hasin M.A. (2021). Fuzzy genetic algorithm-based model for bullwhip effect reduction in a multi-stage supply chain. Int J Supply Chain Inventory Manag..

[6] Lu F., Wang L., Bi H., Du Z., Wang S. (2021). An improved revenue distribution model for logistics service supply chain considering fairness preference. Sustainability.

[7] Zhou X., Bing X., Fei X., Yu L. (2020). Research on quality decisions and coordination with reference effect in dual-channel supply chain. Sustainability.

[8] Loon E.M., Mirjam Brouwers Munnik M., Suzanne Nieuwkerk Pythia, Wouter Curvers, Schoon (2021). Factors influencing health-related quality of life in patients with Barrett's esophagus: a qualitative focus group study. Eur. J. Gastroenterol. Hepatol..

[9] Wang C., Cheng Z., Yue X.G. (2020). Risk management of COVID-19 by universities in China. J. Risk Financ. Manag..

[10] Craighead C.W., Ketchen Jr D.J., Darby J.L. (2020). Pandemics and supply chain management research: toward a theoretical toolbox. Decis. Sci. J..

[11] Viglia G., Dolnicar S. (2020). A review of experiments in tourism and hospitality. Ann. Tourism Res..

[12] Choi N.H. (2021). Analyzing local government capacity and performance: implications for sustainable development. Sustainability.

[13] Sørensen F., Mattsson J., Sundbo J. (2010). Experimental methods in innovation research. Res. Pol..

[14] Williams D.W., Wood M.S., Mitchell J.R. (2019). Applying experimental methods to advance entrepreneurship research: on the need for and publication of experiments. J. Bus. Ventur..

[15] Nazari-Shirkouhi S., Tavakoli M., Govindan K., Mousakhani S. (2023). A hybrid approach using Z-number DEA model and Artificial Neural Network for Resilient supplier Selection. Expert Syst. Appl..

[16] Wu D., Wang Q., Olson D.L. (2023). Industry classification based on supply chain network information using Graph Neural Networks. Appl. Soft Comput..

[17] Li N., Zhu X. (2023). Design and application of blockchain and IoT-enabled sports injury rehabilitation monitoring system using neural network. Soft Comput..

[18] Wang L., Wang Y. (2022). Supply chain financial service management system based on block chain IoT data sharing and edge computing. Alex. Eng. J..

[19] Sawaean F., Ali K. (2020). The impact of entrepreneurial leadership and learning orientation on organizational performance of SMEs: the mediating role of innovation capacity. Manag Sci Lett.

[20] Moeini M., Rivard S. (2019). Responding—or not—to information technology project risks: an integrative model. MIS Q..

[21] Cui L., Wu H., Dai J. (2023). Modelling flexible decisions about sustainable supplier selection in multitier sustainable supply chain management. Int. J. Prod. Res..

[22] Zhang H., He X., Mitri H. (2019). Fuzzy comprehensive evaluation of virtual reality mine safety training system. Saf. Sci..

[23] Wang S.Z. (2020). Trend of competitive sports reform under the background of artificial intelligence. J Phys Conf Ser.

[24] Zhou Y., Li X., Yuen K.F. (2022). Holistic risk assessment of container shipping service based on Bayesian Network Modelling. Reliab. Eng. Syst. Saf..

[25] Teng Y., Wang Y., You H. (2023). The risk evaluation and management of the sports service supply chain by introducing fuzzy comprehensive appraisal and artificial intelligence technology. Expet Syst..

[26] Zhan Y.A., Tan K. (2020). An analytic infrastructure for harvesting big data to enhance supply chain performance. Eur. J. Oper. Res..

[27] Xiong Y., Liu Y. (2021). Research on customer demand acquisition method based on scrap steel reverse supply chain service platform. J Phys Conf Ser.

[28] Salmiah N., Oemar F., Farwitawati R. (2020). Accounting system design for Riau province sports assets: measuring rent system and environment. IOP Conf. Ser. Earth Environ. Sci..

[29] Ma S.C., Kaplanidou K. (2020). Service quality, perceived value and behavioral intentions among highly and lowly identified baseball consumers across nations. Int. J. Sports Mark. Spons..

[30] Taylor N., Livingston M., Coomber K., Mayshak R., Miller P. (2021). The combined impact of higher-risk on-license venue outlet density and trading hours on serious assaults in night-time entertainment precincts. Drug Alcohol Depend..

[31] Li F., Du S. (2023). A quantitative evaluation method for communication impact of sporting events based on SIR dynamic diffusion model. J. Circ. Syst. Comput..

[32] He P., He Y., Tang X., Ma S., Xu H. (2022). Channel encroachment and logistics integration strategies in an e-commerce platform service supply chain. Int. J. Prod. Econ..

[33] Asl-Najafi J., Yaghoubi S., Noori S. (2022). Customization of incentive mechanisms based on product life-cycle phases for an efficient product-service supply chain coordination. Comput. Ind..

[34] Ivanov D., Dolgui A. (2020). Viability of intertwined supply networks: extending the supply chain resilience angles towards survivability. A position paper motivated by COVID-19 outbreak. Int. J. Prod. Res..

[35] Zhang S., Bi C., Zhang M. (2021). Logistics service supply chain order allocation mixed k-means and qos matching. Procedia Comput. Sci..

[36] Guo J., Zhou Y., Li B. (2022). Service-cost-sharing contract design for a dual-channel supply chain with free riding. J. Syst. Sci. Syst. Eng..

[37] Al-Abrrow H., Halbusi H.A., Chew X.Y., Al-Maatoq M., Alharbi R.K., Alnoor A. (2022). Uncovering the antecedents of trust in social commerce: an application of the non-linear artificial neural network approach. Compet. Rev..

[38] Zhang H., Duan D. (2021). Computational ghost imaging with compressed sensing based on a convolutional neural network. Chin. Opt Lett..

[39] Zhang K. (2021). Music style classification algorithm based on music feature extraction and deep neural network. Wireless Commun. Mobile Comput..

[40] Araujo B., Ferreira B., Virtuoso L.S., Meira-Belo L.C., Sebastio R. (2021). A new formulation for polymer fricke dosimeter and an innovative application of neural network to study dose profile from spin-echo NMR data. Radiat. Phys. Chem..

[41] Alshurideh M., Alquqa E., Alzoubi H., Kurdi B., Hamadneh S. (2023). The effect of information security on e-supply chain in the UAE logistics and distribution industry. Uncertain Supply Chain Management.

[42] Ge X., Choi D., Yuan M., Yang Z. (2023). Comprehensive evaluation of high-quality sports industry development in the new era using fuzzy numbers intuitionistic fuzzy sets. J Intell Fuzzy Syst. 2023(Preprint).

[43] Regin R., Rajest S.S., Shynu T. (2023). A review of secure neural networks and big data mining applications in financial risk assessment. Cent Asian J Innov Tourism Manag Finance..

[44] Pham H.T., Pham T., Truong Quang H., Dang C.N. (2023). Supply chain risk management research in construction: a systematic review. Int J Constr Manag..

[45] Luo M., Zhang W., Song T., Bessiere C. (2020).

[46] Luo M., Du B., Zhang W. (2023). Fleet rebalancing for expanding shared e-mobility systems: a multi-agent deep reinforcement learning approach. IEEE Trans. Intell. Transport. Syst..

